# Improving support for breastfeeding mothers: a qualitative study on the experiences of breastfeeding among mothers who reside in a deprived and culturally diverse community

**DOI:** 10.1186/s12939-021-01419-0

**Published:** 2021-04-06

**Authors:** Erica Jane Cook, Faye Powell, Nasreen Ali, Catrin Penn-Jones, Bertha Ochieng, Gurch Randhawa

**Affiliations:** 1 School of Psychology, University of Bedfordshire, Park Square, LU1 3JU Luton, United Kingdom; 2 Institute for Health Research, University of Bedfordshire, Putteridge Bury, Hitchin Road, Luton, UK; 3 Faculty of Health and Life Sciences, De Montfort University, LE1 9BH The Gateway, Leicester, UK

**Keywords:** Breastfeeding, Infant formula feeding, Minority ethnic groups, Deprivation, Qualitative research

## Abstract

**Background:**

The United Kingdom has one of the lowest breastfeeding rates in Europe, with the initiation and continuation of breastfeeding shown to be closely related to the mothers’ age, ethnicity and social class. Whilst the barriers that influence a woman’s decision to breastfeed are well documented, less is known how these barriers vary by the UK’s diverse population. As such, this study aimed to explore mothers’ experiences of breastfeeding and accessing breastfeeding services offered locally amongst a deprived and culturally diverse community.

**Methods:**

A qualitative interpretive study comprising of 63 mothers (white British *n* = 8, Pakistani *n* = 13, Bangladeshi *n* = 10, black African *n* = 15 and Polish *n* = 17) who took part in single-sex focus groups, conducted in local community centres across the most deprived and ethnically diverse wards in Luton, UK. The focus groups were audio-recorded, transcribed and analysed thematically using Framework Analysis.

**Results:**

The most common barriers to breastfeeding irrespective of ethnicity were perceptions surrounding pain and lack of milk. Confidence and motivation were found to be crucial facilitators of breastfeeding; whereby mothers felt that interventions should seek to reassure and support mothers not only during the early stages but throughout the breastfeeding journey. Mothers particularly valued the practical support provided by health care professions particularly surrounding positioning and attachment techniques. However, many mothers felt that the support from health care professionals was not always followed through.

**Conclusions:**

The findings presented inform important recommendations for the design and implementation of future programs and interventions targeted at reducing breastfeeding inequalities. Interventions should focus on providing mothers practical support and reassurance not only during the early stages but throughout their breastfeeding journey. The findings also highlight the need for tailoring services to support diverse communities which acknowledge different traditional and familial practices.

**Table:** 

This article is a part of the Interventions and policy approaches to promote equity in breastfeeding collection, guest-edited by Rafael Pérez-Escamilla, PhD and Mireya Vilar-Compte, PhD	

## Background

Breastfeeding has many advantages for infants and their mothers [1–3] with the health, nutritional, and psychological benefits widely documented [1, 4, 5]. However, despite these considerable benefits, the United Kingdom (UK) remains to have one of the lowest breastfeeding rates in Europe [6]. The World Health Organisation (WHO) recommends that infants are exclusively breastfed for the first six months of life [7]; however, the latest UK Infant Feeding Survey revealed that concerningly only 1 % of infants met this recommendation [8]. The initiation and continuation of breastfeeding have been shown to be closely related to the mothers’ age, ethnicity and social class [6, 8, 9], with highest rates found among older mothers (30+), mothers from Black and Minority Ethnic (BAME) groups alongside mothers who are more affluent and have a higher education status [8, 10]. In the UK the BAME community represent 14 % of the total population [11], compromised of a significant black African and Caribbean (3.3 %) and South Asian (Pakistani, Bangladeshi, Indian) community (7.5 %). More recent migration patterns following the 2004 enlargement of the European Union (EU) have also shown a substantial increase of citizens from Eastern Europe[12]. Whilst there are a range of well-established psychological and socio-cultural factors shown to influence breastfeeding[13–15] there is less understanding of how these vary among the UK’s increasingly ethnically diverse population.

The cultural context in which breastfeeding occurs is important across many levels. Feeding decisions are made in the context of the broader culture which shape our expectations and experiences [16]. Whilst there is a general consensus among the UK BAME population that breastfeeding is best [6]; there are a range of barriers and facilitators within these communities that vary. For example, within Islam breastfeeding is perceived as a spiritual act and a practice of a good Muslim, one which Allah will reward [17, 18]. This is further reinforced through the Holy Qur’an and the Hadith who both recommend that the mother should breastfeed for two years where possible [18]. This will for many Muslim women be an important driver for the initiation and continuation of breastfeeding [18]. However, religious expectations which require women to be covered when breastfeeding can create difficulties in maintaining privacy, particularly given that mothers are more likely to cohabit in large family groups [17]. Exposure, the need for privacy and embarrassment can as a consequence be a distinct barrier for many Muslim women [18–20].

In developing societies, such as Western Africa and Sierra Leone the breast has maintained the primary biological function; an organ used to feed neonates and babies [21]. This has meant that women have been encouraged to expose their breasts in public places freely and without reservations [21], which has in turn normalised breastfeeding. However, developed countries, such as the UK, the cultural context has led to the sexualisation of breasts, with breastfeeding seen as a private act restricted only to neonates [21]. The sexualisation of breasts in westernised societies can often contradict the benefits of breastfeeding [22] and underpins the embarrassment which many mothers feel about breastfeeding in front of others or in public places [23]. Links between acculturation and breastfeeding where higher rates are found in BAME groups who have low levels of acculturation [24–26] and vice versa. Formula feeding among Pakistani communities has also been a symbol of wealth [27] and symbolic of western culture of which they wish to fit in. Therefore, there is a concern that acculturation may lead to a notable reduction in breastfeeding among the BAME community [10, 27].

In some cases, culturally located beliefs have been shown to have an influence on the initiation and continuation of breastfeeding. For example, exclusive breastfeeding among some ethnic minority women might not be practised because of a perception that their breastmilk alone is insufficient for healthy development [22]. The cultural symbolism of weight and health in infants has been shown to increase the use of supplementary feeding [19, 20]. Further, strong beliefs for azan (call for prayers) [28], cultural beliefs such as the colostrum is ‘unclean’ [22, 28] or the perception that breastmilk can be considered stale [18] make it difficult for mothers to initiate breastfeeding immediately after birth. Some of these cultural beliefs could also be seen to encourage early supplementary feeding. For example, there is a tradition in Pakistani culture that the first feed is given by an important family member [29] and date or sweet alternative is given soon after birth for the baby to suck on, a practice called Tahneek [18].

As part of efforts to increase breastfeeding continuation rates, many public and voluntary organisations have delivered additional breastfeeding support services and interventions to provide support in the early postnatal period and beyond. However, it is unclear to what extent these services are culturally tailored to ethnically diverse populations. Some minority ethnic women reported language difficulties and feeling afraid or unable to challenge nurses when they were unhappy with the ward practices or care [22]. Barriers to the promotion of breastfeeding perceived by health professionals include latent hostility in the local community towards breastfeeding and lack of staff breastfeeding knowledge [6].

Whilst the barriers that influence a woman’s decision to breastfeed are well documented, less is known on how these barriers vary across and within different ethnic groups who reside in the United Kingdom. Given that the diverse UK population [11] there is an increased need to ensure that interventions to support breastfeeding are tailored for our diverse population and importantly do not add to the existing health inequalities than many BAME communities face. In order to develop and implement effective breastfeeding interventions, it is essential to better understand the knowledge and experience of mothers from broad socio-demographic groups and how this influences their behaviours. As such, this study aims to explore mothers’ experiences of breastfeeding alongside the barriers toward accessing breastfeeding services offered locally amongst a deprived and culturally diverse community.

## Methods

This study used a qualitative interpretative research design using focus group discussions to generate in-depth contextualised information from a range of opinions and experiences.

### Sampling

The site for this study was Luton, an ethnically diverse town located in the South East of England. Luton is only one of three towns in the UK to have a white British population of less than 50 %; serving a large South Asian (Pakistani, Bangladeshi) (21.1 %), African-Caribbean population with a more recent increase of residents from Eastern Europe most commonly Poland [30]. Luton also experiences high levels of deprivation across all domains when compared to other parts of the UK [31] where larger sections of the visible BAME community reside.

Wards which are electoral boundaries used to divide a town or city into smaller geographies were used as a basis to recruit participants. Each ward hosts an average population of around 6,600 [32] and can be a useful geography when targeting specific communities. As such participants were recruited from the five most deprived wards across Luton(as identified by the Index of Multiple Deprivation 2015 [33]). The sociodemographic characteristics of these five wards are presented in Table 1.

**Table 1:** Socio-demographic characteristics of chosen wards

WARD	Socio-demographic characteristics
**1 and 2**	Higher proportion of non-white; mainly Pakistani and Bangladeshi ethnicity. This ward is predominantly of Muslim religion.
**3**	Higher proportion of lone parent families with dependent children when compared to the Luton/UK average. This ward has a predominantly White population with a recent high influx of Polish immigrants.
**4**	This ward has a higher proportion of Black African, Caribbean and White British residents compared to the rest of Luton. This ward is predominantly Christian or non-religious.
**5**	This ward has a higher percentage of White Europeans (e.g. Polish), Black African compared to the rest of Luton. This ward is predominantly Christian or non-religious.

Purposive stratified sampling was used to recruit mothers of at least one child aged 0–5 years old, who were aged between 21 and 45 years old. Those eligible to take part had to self-identify as being from one of the five target wards, as well as one of the following ethnic groups: white British, South Asian (Pakistani and Bangladeshi), black (African/Caribbean) or Polish. The ethnic group categories were based on the Census (2011) [11] for the purpose of this study. All participants regardless of fluency of English were included in the study to ensure that we captured the views of a range of mothers, enabled through the use of bilingual interviewers fluent in Polish, Punjabi, Pahari (dialect of Punjabi) and Urdu.

To achieve a representative sample a systematic multi-pronged approach was taken to recruit participants. Firstly, advertisement material and information about the study was sent via e-mail directly to managers and/or researcher contacts across all community centres within the defined wards. It is important to note that the research team have extensive community networks across the local area, and these were utilised where possible. Posters and other recruitment material including leaflets were widely advertised throughout the wards, displayed and handed out in local community settings including local amenities, children’s centres, community centres and places of worship. Facilitators also recruited participants face to face through attending local community groups and places of worship with permission of community leaders. If prospective participants were interested in taking part their details were then passed onto the research team who then contacted them to ensure they met the eligibility criteria, to discuss the study and answer any questions. Those eligible were then contacted by the facilitator at a later date to invite them to attend a focus group discussion.

### Data collection

A semi-structured, open-ended topic guide was developed collaboratively by the research team who are all senior researchers with expertise in nutrition, health inequalities and eating behaviour. The topic guide was used as part of a larger body of work, exploring several topics on nutrition and eating behaviour. Questions concerning the feeding practices from birth to early weaning were open, with probes to elicit a wide range of discussions. In particular, questions asked focused on feeding method, the influencers of this decision, and experiences of adopting this feeding method, including any intervention services accessed (Table 2). The topic guide alongside all study materials were pretested by EC and FP with a sample of undergraduate diverse female students (whom were all mothers) to check if the questions were clear, culturally relevant and to identify any issues. No amendments were needed.

**Table 2:** Topic Guide Questions

*I would like to ask you a little bit about feeding your child from birth through to weaning.*	
*• What feeding method did you choose for your new-born baby?*	
*◦ Probe: whether they breast fed and or/formula, exclusively breast feeding or combined, expressed the breast milk, duration.*	
*◦ Probe: child’s response to feeding method.*	
*• Who/what influenced your decisions about feeding your child/children?*	
*◦ Probe: for breast feeding vs. formula feeding, family, friends, internet, TV, radio, midwives, health visitors, cost, availability, culture and religion.*	
*• What was your (and your wife’s/partner’s) experience of feeding your child/children?*	
*◦ Probe: for breast feeding vs. formula feeding, difficulties and easy/good. If received support/advice or not, who was support/advice from.*	
*• What services/interventions did you access to support you and what were your experiences of accessing these*	
*◦ Probe: Breastfeeding clinics, healthcare professionals, other*	

There was a total of 13 focus groups with mothers (n = 63) aged between 21 and 45 with all focus groups organised by the five ethnic groups identified and age (21–30; 31–45).

Identifying a suitable sample size for focus group discussions is a contentious issue. For example, whilst a sample size of six to eight is generally accepted as a sufficient for a focus group discussion [34] others have argued that smaller sample sizes are sufficient i.e. four to eight [35, 36]. The research team were mindful to organise focus groups that were large enough to generate rich discussion on the aforementioned topics, but not too large that some participants would lose their voice in the discussion.

The majority of focus groups achieved a sample size of four participants or above (*n* = 9, 70 %; see Table 3). However, for some focus groups there were some notable challenges with the recruitment of mothers. The main issue related to non-attendance (Bangladeshi *n* = 2; Polish *n* = 3; white British *n* = 3; black African *n* = 2) with no reason given, a common issue found in focus group research. There were also a few mothers across the focus groups who dropped-out prior to the focus group commenced due to childcare and/or work responsibilities (Bangladeshi n = 1; black African n-4).

The number of focus groups was solely driven by saturation, i.e. the point where consecutive focus groups revealed no additional second-level categories [37]. Saturation across the sample was normally found at around two focus groups; however, in situations where focus groups were unable to recruit more than four mothers then additional focus groups were held to ensure that saturation was reached. For example, for black African mothers four focus groups were held and Polish mothers three focus groups were held. A breakdown of the focus groups and demographic information about the participants (employment status, education level and marital status) are provided in Table 3. Fathers whilst included in this study their findings are not presented in this paper.

**Table 3:** Sample breakdown for all focus groups

Focus group	Ward	Ethnicity	Gender	Age	*N*	Marital Status	Occupation status	Education level^a^	Duration (Mins)
1	1	Pakistani	Female	31–45	8	Married (8)	Unemployed (8)	Secondary qualification/s (3) Undergraduate degree level (4) 1 x University entry level qualification/s	91
2	1	Bangladeshi	Female	21–30	5	Married (4) Divorced (1)	Unemployed (1) Full-time education (1) Part-time employment (2) Not specified (1)	Undergraduate degree level (1) University entry level qualification/s (4)	85
3	2	Pakistani	Female	21–30	5	Married (5)	Unemployed (5)	University entry level qualifications (5)	73
4	2	Bangladeshi	Female	31–45	5	Married (5)	Unemployed (3) Full-time employment (2)	University entry level qualification/s (4) Secondary qualification/s (1)	78
5	3	White British	Female	21–30	4	Single (2) In a relationship (2)	Unemployed (4)	Secondary qualification/s (4)	79
6	3	White British	Female	31–45	4	Married (1) Single (1) In a relationship (2)	Full-time employment (2) Part-time employment (1) Self-employed (1)	Secondary qualification/s (2) Undergraduate degree level (2)	75
7	4	Black African	Female	31–45	2	In a relationship (2)	Full-time employment (2)	Undergraduate degree level (1) Secondary qualification/s (1)	72
8	4	Black African	Female	21–30	8	Married (2) In a relationship (4) Single (2)	Unemployed (5) Part-time employment (3)	University entry level qualification/s (2) Secondary qualification/s (6)	88
9	5	Black African	Female	31–45	2	Divorced (1) Single (1)	Full-time employment (2)	Secondary qualification/s (2)	82
10	5	Black African	Female	31–45	3	Married (1) In a relationship (2)	Full-time employment (3)	Secondary qualification/s (3)	81
11	5	Polish	Female	26–37	8	Married (7) In a relationship (1)	Unemployed (8)	Secondary qualification/s (3) University entry level qualification/s (2) Undergraduate degree level (1) Masters level and above (1)	83
12	5	Polish	Female	33–39	6	Married (6)	Full-time employment (6)	No formal qualification (1) University entry level qualification/s (5)	77
13	5	Polish	Female	27–35	3	Married (3)	Unemployed (2) Full-time employment (1)	University entry level qualification/s (3)	72

^a^Secondary qualifications (GCSE or equivalent), University entry qualification (AS/A level or equivalent), Post-secondary below degree level (HNC/HND, NVQ levels 4 and 5, BTEC higher etc.), Undergraduate degree level (university), Masters level and above (higher degree, doctorate).

Focus groups lasted approximately 90 min and took place in locations convenient for participants, typically community centres. Focus group methodology was chosen on the basis that it can provide an invaluable approach particularly when conducting research in cultural diverse communities [38] and through the generation of group interactions can providing interesting, rich, and complex data which can help participants to explore and clarify their views in ways that may be less accessible in one to one interviews [28, 29].

All focus groups were carried out by trained female and where appropriate bilingual facilitators who were recruited by the research team from the University of Bedfordshire. All facilitators held a postgraduate qualification in health sciences and were experienced qualitative fieldworkers. They were all required to complete a one-day training event delivered by the research team which provided an overview of the research study, presented the research instruments to be used and provided the facilitators an opportunity to practice the topic guide with trained researchers. All focus groups with Polish mothers were conducted in Polish supported by a bilingual facilitator. Focus groups with Pakistani and Bangladeshi mothers were facilitated by bilingual facilitators proficient in Punjabi, Pahari and Urdu which allowed for rapport generation and inclusion of non-English speakers. Most South Asian focus groups were facilitated in English with some explanations in the vernacular. Black African and white British focus groups were all conducted in English by female ethnic matched facilitators. It is important to note that participants did not know facilitators prior to participation and focus groups were not attended by anyone outside of the facilitator and participants.

A participant information sheet and consent form were provided, and participants were afforded the opportunity to ask questions. These detailed the nature and purpose of the research, including the principles of anonymity, confidentiality and that participants could withdraw at any time without reason. Focus groups were audio recorded, these recordings were stored anonymously with access only provided to FP and EC. Following the focus group, a £10 high street voucher was given to participants to compensate for their time and travel costs to attend.

### Analysis

Focus groups conducted in English were transcribed verbatim with Polish, Punjabi, Pahari and Urdu back translated into English by the bilingual facilitator which were then validated by the research team. All transcripts were analysed using the Framework Analysis Approach (FA) [39]. FA is a grounded and generative analytical procedure which uses distinct connected stages [40, 41] which involves detailed familiarisation with the data, identification of key themes, and interpretation of the findings within the context of other research as well as policy and practice considerations [42]. FA facilitates the analysis through a matrix containing data summaries, in particular allowing comparisons to be made between themes and cases or participants, which allowed the research team to explore feeding practices between ethnic groups.

Transcripts were reviewed (by EC and FP) to ensure they accurately represented the audio and to generate written notes and reflections. Using NVivo 11, preliminary coding was conducted on each line of the transcript, and initial codes and impressions were generated based on these, as well as reflective notes described previously (by EC and FP). Three transcripts were then coded independently and reviewed by EC, FP and CP-J, with coding agreement discussed and the generation of an initial coding matrix. This matrix was applied to two further transcripts to ensure it captured all relevant themes and was adapted to ensure new codes or themes were included. An agreement was sought from the research team on the full matrix, which was then applied to the entire data set. The matrix was then reviewed and synthesised to develop final themes grounded in the data, key dimensions were identified, and comparison between cases conducted accordingly. These core findings were also discussed with interviewers.

## Results

Themes and sub-themes were organised around the barriers and facilitators of breastfeeding (see Table 4). Barriers towards breastfeeding resulted in four main themes, which included (1) physical barriers; (2) psychological barriers (3) embarrassment of breastfeeding in public and (4) time and convenience. Facilitators centred around five themes (1) cultural traditions and practices; (2) positive perceptions towards breastfeeding; (3) flexible approaches to feeding; (4) perseverance and (5) support from healthcare professionals and breastfeeding services. These themes and their resulting subthemes are discussed in detail below.

**Table 4:** Analytic coding framework with definitions

Barriers to breastfeeding	
**CODE**	**DESCRIPTION**
**Physical barriers**	
Pain during/after breastfeeding	*Pain and/or soreness associated with latching, positioning, engorgement, biting/teething of infant, medical reasons/other whilst breastfeeding either initiating/during/after breastfeeding/expressing milk.*
Lack of milk	*Perceptions of lack of milk, weight gain of infant, failure to thrive, sleep patterns, strategies to address milk supply issues.*
Illness and medical conditions	*Pre-existing and/or new medical conditions/infections/illnesses/medications that impact ability to breastfeed, medical advice to stop/reduce breastfeeding.*
**Psychological Barriers**	
Stress and coping with breastfeeding	*Perceptions of stress towards breastfeeding, coping strategies and mechanisms to address levels of stress, impact of stress on mother/infant/other.*
Sense of failure to breastfeed	*Guilt, blaming self, disappointment, remorseful, depression, impact on self/attachment with infant/others from not initiating or stopping breastfeeding.*
Pressure from important others	*Feeling pressured to introduce formula, cease breastfeeding from partners/husbands, immediate family (i.e., mothers, mother-in-law), healthcare professionals, friends, other for health/emotional reasons.*
**Embarrassment**	
Stigma of breastfeeding in public/in front of family	*Embarrassment, feeling uncomfortable to breastfeed in front of others (public/ friends/family/other), fearful of other’s reactions to breastfeeding, disapproval from others (public/family/friends/other).*
**Time and convenience**	
Competing responsibilities	*Managing competing responsibilities including other children/childcare/other caring responsibilities, work, household chores, time for self/partner/other.*
Time to breastfeed	*Expressing, time taken to latch/feed, duration of feeding and impact this has on daily life.*
Convenience of bottle feeding	*Convenience and ease of bottle feeding (formula) versus breastfeeding at home/public/work/other, sharing responsibility of feeding, reduced pressure to be sole person to feed infant.*
**Facilitators to breastfeeding**	
**CODE**	**DESCRIPTION**
**Cultural traditions and practices**	
Cultural traditions and expectations related to breastfeeding	*Attitudes, beliefs and behaviours of important others and those around you, influential traditions, practices and cultural values towards breastfeeding, normalisation of breastfeeding.*
The role of religious beliefs in breastfeeding	*Religious beliefs, maternal religion, religious endorsement of breastfeeding, religious beliefs of the importance of breastfeeding.*
**Positive perceptions towards breastfeeding**	
Perceived benefits of breastfeeding	*Perception of advantages of breastfeeding for mother/infant/other includes physical and emotional health, bonding and attachment.*
Perceived importance of breastfeeding	*Value and worth of breastfeeding to infant/mother/other (short and longer term).*
**Perseverance**	
Motivation to breastfeed	*Willingness to breastfeed, a desire to breastfeed and succeed in breastfeeding.*
Confidence to breastfeed	*Belief in own ability to breastfeed and/or overcome difficulties and obstacles to breastfeed, determination, empowerment.*
**Flexible approaches to feeding**	
Involvement of fathers/others in breastfeeding	*Involving fathers/others in feeding infant, expressing, sharing breastfeeding/breastmilk among mothers.*
Division of responsibilities to feeding	*Sharing responsibilities of feeding, care for infant.*
**Support from Healthcare Professionals/ Breastfeeding services**	
Practical advice, support and reassurance	*Types of services/support accessed related to infant feeding/breastfeeding, healthcare professional led, peer led*, *Mode of delivery (face to face, telephone, online), adherence to advice.*
Relationship with healthcare provider	*Experience with healthcare professional, rapport, quality of relationship, quality of communication, trust, support.*
Continuity of care	*Mothers perception of quality of care across time, level of follow up and longer-term support provided, ongoing relationship with healthcare professionals and/or services*,
Consistency of advice	*Consistency of breastfeeding advice across healthcare professionals and/or services.*
Access to support and breastfeeding services	*Knowledge and awareness of services, barriers to access, ease of access, cultural sensitivity.*

The majority of mothers irrespective of age and ethnicity had made the decision to breastfeed prior to birth. It was common among all focus groups that decisions regarding the uptake and continuation of bottle or breastfeeding were made by the almost entirely by mothers and that this decision was not influenced by the father. This was particularly common amongst South Asian groups.

there was no contribution regarding this from my husband’s side to be honest (laughter)…the previous child that we had, she didn’t just latch at all. And he saw obviously the difficulty I had, so he said that whatever decision you are making I am not going to have any impact on it

*(Pakistani female, aged 21–30)*.

### Barriers to breastfeeding

#### Physical barriers

Pain and perceptions regarding lack of milk were common barriers discussed by mothers across all groups irrespective of ethnicity. Perceptions surrounding *lack of milk* were prevalent across South Asian (Pakistani and Bangladeshi), Polish and white British focus groups. There was a perception across many of mothers that their babies did not have sufficient milk from breastfeeding, which led to the decision to introduce formula. These perceptions were based on one or more of the following reasons: infant not sleeping for long periods, infant not gaining as much weight as expected and/or perception that their infant was more content when receiving bottle/formula. Weight concerns were, particularly common among South Asian mothers particularly when their baby had a low birth weight and were not thriving.

He was a tiny baby when he was born, I thought he would gain weight if breastfed, but the weight kept decreasing so I gave formula milk. He seemed satisfied with formula and started gaining weight

*(Pakistani mother, aged 31–45)*.

Breastfeeding wasn’t enough for her [infant] so I had to top her up with formula. We were worried because she was small when she was born. She would cry every thirty minutes for me but then didn’t like me going near her, she just got frustrated

*(Bangladeshi mother, aged 31–45).*

In terms of addressing this there were limited strategies discussed; however, a few South Asian mothers were given additional foods such as Panjiri (a traditional sweet, made with a base of ghee, whole wheat flour, and dried sugar) which is said to make the mother ‘bigger’ so that they were able to produce more milk. One (Pakistani) mother also mentioned that she was told to express more to increase supply. Some mothers (Polish and South Asian) who were supplementing breastfeeding with expressed milk were also concerned that the amount they were expressing was too low to sufficiently meet their child’s needs, as discussed by one Polish mother ‘*Well my husband was very terrified when he saw how upset I was about having too little milk and that I should have more****[choking up during statement]***, *because we started to pump the milk and why is the child always hungry and then it turned out that he was constantly hungry because I was pumping 20 ml of milk and not the 150 ml. that he needed, so then, my husband was terrified with the state of my mentality and that I am terrified/worried, yes, with that situation more than about the situation rather than feeding the child.’* (Polish mother, aged 31–45). As such mothers liked the reassurance of giving their infant formula and knowing the quantities of milk they have received *‘I felt comfortable with formula feeding that my child was filling up’* (Pakistani mother, aged 21–35).

*Pain* was also a concern raised by some white British and Polish mothers.

I would just like to say that I think it [breastfeeding] was the worst experience ever, the worst that could possibly be my nipples bled, it hurt, I couldn’t cure it, it was a disaster; it was the worst experience of my life…..worse than actual birth.

(Polish mother, aged 31–45)

A few mothers, mostly Polish and white British mentioned that *illness and medical conditions* influenced their decision to either not initiate breastfeeding or terminate early. It was not clear in most situations if this decision was based on a consultation with a healthcare specialist or if this was a personal decision based on their judgement.

Gutted that I couldn’t do it for my first. I expressed for my second in hospital but I got mastitis and so I stopped

(White British mother, aged 31–45)

### Psychological barriers

Many mothers discussed the stress and inability to cope with not being able to breastfeed. There was an overwhelming sense of failure associated with not being able to breastfeed, which was also discussed particularly among Polish mothers in relation to the preconception of running out of milk.

well, it wasn’t that I physically could not cope with his demand, but psychologically I couldn’t cope any longer and I couldn’t cope with thinking anymore of what am I going to do when I run out of milk and well I was a bit upset about this

(Polish mother, aged 21–30)

On the contrary, my mother said that it’s not true and that I should not stress myself out over it so much, because I was bottle-fed and I’m still alive and something and that in the end finally someone said something intelligent that I shouldn’t wind myself up about it and get frustrated, because it was a big thing for me, and that if I am to get so worked up over it [breastfeeding] then I should just not do it.

(Polish mother, aged 31–45)

Some mothers felt the pressure of others was a psychological barrier to continue breastfeeding. For example, concerns regarding weight gain were particularly prevalent among South Asian mothers who often felt pressured by others (husband, mother, sisters) to give their infant formula to solve this problem. One Bangladeshi mother recalled her experience of birth following a c-section. She did initiate breastfeeding, but her baby dropped weight at the beginning. She discussed her concerns with both her sister and her mother who advised her to give the child the ‘bottle’ (formula). Whilst she valued their opinions, she wanted to succeed in breastfeeding, so she felt that this added additional pressure put additional stress on to her.

### Embarrassment of breastfeeding in public

One barrier discussed among white British mothers was the embarrassment and stigma associated with breastfeeding. Many mothers felt that they were too embarrassed to breastfeed either in public or in front of their immediate family (i.e., parents, in-laws). Some mothers discussed the process of adjusting to this, but others found it more challenging and felt more comfortable bottle feeding.

To be honest I am a bit of a prude, I just found it really embarrassing I didn’t even want my parents to come to hospital when breastfeeding

*(White British mothers, aged 31–45)*

Breastfeeding is stigmatising. I found breastfeeding in public and in front of in laws as scary but now I don’t care.

*(White British mothers, aged 21–30)*.

### Time and convenience

Pakistani and Bangladeshi mothers discussed the challenges of managing household tasks as well as looking after more than one child ‘*Can’t do anything else around the house while breastfeeding directly, so I’m expressing so someone else can feed child. Consuming a lot of time getting him to latch on, and expressing milk takes time. It is just really difficult to manage’* (Bangladeshi mother, aged 21–30). This issue was not discussed amongst any other ethnic groups.

There were also discussions among South Asian mothers about privacy and breastfeeding with a preference to bottle feed in public. Many mothers sometimes expressed milk, but this was viewed as too time consuming. Other mothers who did breastfeed when out in public discussed having to breastfeed their child at home before they went out or breastfed in the car before they got out to go shopping. Whilst breastfeeding was acknowledged to be better for the baby formula feeding was viewed among many of the mothers as a much more convenient option.

**M1: ***‘It is hard to breastfeed when you go out. You have to find somewhere private, so it takes more time’*.

**M2**: *‘Yes, I struggle to breastfeed in public so do it in car or keep a bottle just in case’***M1**: *‘It give you options, powdered milk 'oh it's alright'’ (Bangladeshi mothers, aged 21-30)*

Mothers also discussed the advantages of bottle feeding allowing more *shared responsibility* in feeding. Using formula allowed the fathers to take a more active role in feeding their infant and would be able to support the mother.

*‘I believe that using formula is all the better, because it makes it all the easier for the man to help with feeding, because he can make the milk and oh, let’s say that he will get up in the middle of the night and then in that instance he prevents us from getting up in the middle of the night, because he can just go on his own and do it and the child is happy and that we don’t have to get up and give [food] to the child’.*

*(Polish mother, aged 21–30)*.

### Facilitators

#### Cultural traditions and practices

Many mothers discussed the importance of previous of cultural traditions in their decisions regarding the uptake and continuation of bottle or breast feeding. For many mothers their family’s tradition influenced their decision to breastfeed.

well, it’s sort of a tradition [my] mother [breast]fed, so I [breast]fed'

(Polish mother, aged 31–45)

All black (African) mothers we interviewed breastfed, with many feeding their infant for over two years. African mothers discussed that within their culture, breastfeeding was very much valued, where it would be common for mothers to breastfeed for two years or more. For South Asian mothers (Pakistani and Bangladeshi) religion (Islam) was the most important influence on their decision to breastfeed. Islam was seen to encourage breastfeeding, with many disclosing that they are culturally expected to breastfeed for at least two years ‘*Islam encourages two years for breastfeeding. Religion influences more than anything else’* (Pakistani mother, aged 21–30). Benefits of breastfeeding from an Islamic perspective cantered on enabling the mother and baby to share a close mother-infant bond. One respondent also expressed that breastfeeding *‘makes you feel like you are serving someone else, like Allah it is a blessing’* (Bangladeshi mother, aged 31–45). Many mothers disclosed that for cultural reasons they delayed feeding their infant for three days after they were born which was said to be related to the quality of their milk. However, many of the mothers felt that this may have negatively impacted on their milk supply and made it more difficult for them to establish a good latch technique with their baby.

Female relatives (mothers, mother-in-law, sisters, sister-in-law) were a significant influence particularly among South Asian, Polish and black mothers who were viewed to provide an important role model which they wanted to emanate. Breastfeeding was very much normalised within their family and many participants recalled experiences of watching those around them breastfeeding. This was however, in contrast to white British Mothers where family appeared to have less influence on their decision *‘Mum, sister and sister-in-law didn’t breastfeed, so I was nervous, they all seemed surprised that I wanted to do it’* (White British mother, aged 31–45).

### Positive perceptions towards breastfeeding

Black, South Asian and Polish mothers held the most positive beliefs towards breastfeeding. The benefits of breastfeeding shared among black respondents centred on improved attachment (bonds the mother with the baby) alongside physical health and strength of the infant (improves the body and build of the child, increases strength, reduces ill health) as one mother states ‘*don’t see sickness in breastfed children they are strong. Mothers should give their breastmilk to children like cow gives their milk to children’* (Black African mother, aged 31–45).

The benefits of breastfeeding among South Asian mothers were similarly centred around attachment (improves bond between mother and baby) alongside improving physical and psychological health ‘*My second two children didn’t take but my youngest breastfed. I think the children who were breastfed are more mentally and physically active than second two because they weren’t breastfed’* (Pakistani mother, aged 31–45). Benefits for the mother was also discussed including improved weight loss, improved mood (reduces postnatal depression), increase of maternal instincts. Polish mothers also had positive attitudes towards breastfeeding; however, their disclosed benefits were more centred on the physical health of the infant (improved nutrition, improved health for child, less infections and illness for child).

However, white British mothers who were the least likely to breastfeed did not outline any benefits in relation to breastfeeding for either the baby or the mother. They also held the most negative views (particularly younger mothers) towards breastfeeding across all ethnicities *‘I hated breastfeeding and expressing milk. I just don’t like it’* (White British mother, aged 21–30).

### Perseverance

Many mothers mentioned the importance of having the motivation and confidence to persevere. Mothers also mentioned the need to focus on the reciprocal nature of breastfeeding, a journey for you and baby with a focus on the baby learning rather than mum at fault.

*‘and I know I decided very early on that was going to breastfeed, so for me it was, I already decided that I must do it, I am going to persevere, and then it really hurt there was lots of bleeding and then eventually it was fine [laughter] she, I reckon she just worked it out [laughter] no matter what I was doing wrong she just went ‘ok I’ve figured it out mum you can calm me down now’ [laughter] [F1: ‘I’ve done it!’] yeah*.

*(White British mother, aged 31–45)*.

### Flexible approaches to feeding to involve fathers & ‘share the load’

Polish mothers discussed using flexible approaches to feed their infant with the addition of expressed milk so that they could ‘share the load’ and divide responsibilities between them. Polish mothers seemed to have more open and flexible ideas about feeding, with some mothers discussing sharing breastfeeding with other mothers.

she always associated him with that fact that it will be bottled milk and associated myself with the breast and on, and on that basis that is how she consumed the milk. He didn’t have any difficulties, in particular he didn’t have any difficulties doing it this way in my case.

*(Polish mother, aged 21–30)*

With us it was the same situation, uh it was the case that my partner had helped and so the duties were divided.

*(Polish mother, aged 21–30)*.

### Support from Healthcare Professionals/ Breastfeeding services

Many South Asian and white British mothers talked a lot about the importance of the help and support they received from local health care professionals/breastfeeding services. Many mothers discussed the struggles that they had at the beginning including feeding and latching problems and felt that the additional practical advice and support was what encouraged them to persevere *‘Once I went to [name removed] breastfeeding clinic…My baby wasn’t taking breast milk at all….They told me how to hold the baby and how to help the baby to latch on. I didn’t know all this before that’* (Pakistani female, aged 31–45). Older white British mothers also valued being signposted from their midwife to services that were able to support them including; lactation consultations, drop in feeding clinics and breastfeeding cafes.

There were some discussions regarding lack of support among some of the (Bangladeshi) mothers *‘There is a lack of help in area when you haven’t got enough breastmilk’* (Bangladeshi mother, aged 21–35). There were also discussions surrounding (lack of) continuity in care with a general consensus that support whilst well received was not always followed up. For example, one breastfeeding mother discussed going to see a breastfeeding nurse for help on how to latch her baby but when she got home, she found it hard to put what she learnt into practice so she would have liked some follow up support and reassurance that she was doing it right. Further, some mothers disclosed that they felt more alone as their infants got older as the support moved from a home care to a community setting.

Younger white British Mothers were also more critical of the support from healthcare professionals. There was some discussion around conflicting information particularly within hospital settings *‘In hospital I had six different people telling me different ways to breastfeed’* (White British mother, aged 21–30). Also, some younger white British mothers discussed feeling judged by some healthcare professionals and felt that ‘discussions’ around breastfeeding were very officious rather than supportive *‘She looked at me like a young mum and drilled into me about breastfeeding but then there was no support after. Advised on when to feed kid, don’t do this, don’t do that “well what is there anything I can do with my child?’* (White British Mother, aged 21–30). Discussions also revealed that they felt judged for not being able to breastfeed *‘It is the worst feeling ever not being able to breastfeed, but they [healthcare professionals] think you should be able to as a mother. She just looked at me like a young mum who couldn’t be bothered to breastfeed’* (White British mother, aged 21–35). An important point to also note is that whilst many Polish mothers discussed their challenges in breastfeeding, both emotionally and physically there was no discussion of accessing any support.

## Discussion

The findings from this study provide a current qualitative exploration of the barriers and facilitators of breastfeeding among mothers who reside in a culturally diverse and deprived community. The findings revealed a range of themes related to barriers of breastfeeding which included (1) physical barriers; (2) psychological barriers (3) embarrassment of breastfeeding in public and (4) time and convenience. Facilitators of breastfeeding centred around (1) cultural traditions and practices; (2) positive perceptions towards breastfeeding; (3) flexible approaches to feeding; (4) perseverance and (5) support from healthcare professionals and breastfeeding services. These themes are discussed in detail below.

The most common barrier to breastfeeding disclosed among South Asian, white British and Polish mothers surrounded perceptions that their breastmilk was not sufficient to sustain the child which led to the decision to introduce formula to meet their child’s perceived demands. This was particularly pertinent among South Asian mothers, a finding consistent with previous research [27, 43]. The barriers of prolonged breastfeeding practices particularly among those who have an infant with a low birth weight have been widely documented, shown to particularly among mothers of lower maternal age and education, and those of non White ethnicity [44, 45]. The findings revealed that the mothers fears were often reinforced through making comparisons to their often-low expressed milk output alongside their babies perceived contentment when receiving formula. Whilst lack of milk can often be a perception rather than reality, the strategies such as early initiation of expression and increased frequency of expression can increase breastmilk production particularly in low birth weight infants [46]. However, despite these concerns very few mothers we interviewed discussed any strategies that they had used to overcome this. Therefore, more support and reassurance particularly to mothers with low birthweight infants on how to manage perceived or real issues related to low milk supply, would appear to be very welcome. Myths and misconceptions surrounding milk supply should also be addressed and would perhaps be better timed prenatally.

Confidence and motivation were shown to be key facilitators of breastfeeding; whereby mothers felt that interventions should seek to reassure and support parents not only during the early stages but throughout the whole breastfeeding journey. Positive attitudes and the strength of these beliefs surrounding breastfeeding were most pertinent to those who not only initiated breastfeeding but successfully continued to breastfeed. Whilst black African, South Asian (Pakistani and Bangladeshi) and Polish parents all held positive attitudes towards breastfeeding, the beliefs varied, a reflection of cultural and religious differences. The normalisation of breastfeeding and the value it holds within the mother’s culture was found to be an essential factor in influencing these beliefs. Therefore, when framing interventions to promote the benefits of breastfeeding it is important that messages are culturally tailored with a focus on the psychological benefits of breastfeeding as well as the physical [19].

Many parents among our diverse sample felt that their traditional cultural and religious beliefs surrounding breastfeeding contradicted those held within the UK. South Asian (Pakistani and Bangladeshi) and Polish mothers for example were found to hold positive views towards the use of formula feeding, mostly as an alternative to overcome barriers (lack of privacy, ease and convenience), a finding consistent with previous research particularly within communities with high levels of social deprivation [47, 48]. Research has shown that the status of formula feeding across diverse low-income communities is often reflective of being a ‘modern’ mother which can influence a culture towards early cessation or mixed feeding practices [48]. The impact of acculturation, alongside the conflict of cultural beliefs and practices of breastfeeding and meeting the perceived needs of their infant whilst residing within a formula feeding culture have all been shown to be important factors that govern feeding decision making [27]. This finding therefore reinforces the increasingly important role that acculturation plays alongside the normalisation of artificial feeding on feeding decision processes, with evidence that this may also extend to more recently migrated population groups within the UK.

Many mothers valued practical support provided from health care professions particularly surrounding positioning and attachment techniques. The access to support appeared to vary across ethnic groups and locality. For some, there was ample support ranging from breastfeeding clinics, drop-in clinics, and breastfeeding cafes for others this was less evident. This was particularly a concern for Polish mothers who despite having substantial struggles through their breastfeeding journeys did not discuss accessing any support. Eastern European migrants reportedly have lower uptake of healthcare services, alongside a widespread preference for transnational care [49] i.e. the desire to give and receive care or support across borders, often used to maintain close, continual ties to their country of origin [50]. Lack of knowledge regarding services alongside differing patient expectations are core barriers to access, particularly among those unable to speak English [51]. Therefore, it is essential that health care providers ensure that breastfeeding services are culturally tailored to more recently migrated communities, with services to remain flexible to adapt to migrants’ changing needs and preferences.

Young white British mothers in our sample found breastfeeding particularly challenging, a finding consistent with previous research particularly within deprived low-income communities [52–54]. Many young mothers felt uncomfortable around the idea of breastfeeding which many perceived as unnatural. The social hostility of breastfeeding in public and sexualisation of breasts have been found to contradict the well-publicised benefits of breastfeeding [22]. Discussions revealed that many of these mothers had no personal experience of breastfeeding and felt that artificial feeding was not only normalised but advocated by those around them. Therefore, interventions which focus on creating an environment where breastfeeding is not only accepted but encouraged will support and encourage younger mothers to have more positive attitudes towards breastfeeding practices [54]. Interactions with healthcare professionals were also viewed negatively. There were perceptions that as a younger mother they experienced formula feeding stigma through interactions with healthcare professionals, where they were stereotyped to have made the decision to feed artificially. Perceptions regarding formula feeding stigma has been a common finding particularly among younger mothers from low-income communities [55]. Giving our findings and impact these perceptions can have on the mothers relationship with healthcare professionals highlights the importance for more inclusive discussions surrounding breastfeeding to take place, focusing on the development of positive and reciprocal dialogue to allow mothers the opportunity to discuss their hopes and fears.

### Strengths and limitations

This study compliments the existing research base through the inclusion of Polish perspectives on infant feeding and diet. Polish migrants in the UK are estimated to number 832,000 as of 2015, and Poland is the most common non-UK country of birth [56], therefore the inclusion of this group supports the prevalence of this community within the UK. In addition, the separation of South Asian ethnicities into Bangladeshi and Pakistani, allowed for exploration between these groups that are often treated in a homogenous manner, when in fact culturally there are considerable differences between them. Also, the use of ethnically matched bilingual facilitators contributed to the depth of the focus group discussions. Finally, previous scholars have discussed engaging with socially disadvantaged groups as challenging, therefore this study as well as recruiting an ethnically diverse sample, who could express themselves in the language they were most comfortable with, also provides perspectives from under researched deprived groups [57].

However, there are some limitations to this work. Although the diversity of this sample is a strength, challenges in recruiting black Caribbean females were found. Further, although the sample is broad in its inclusion, it is not exhaustive of all ethnic groups residing in Luton UK. Therefore, it is important when applying these findings, that the omission of these groups is noted. It should also be acknowledged that all participants whilst had at least one child aged 0–5, those who had a child who was older may have experienced issues recalling their experience during breastfeeding. As a result, these participants may be more influenced by group dynamics due to this recall bias. The Framework Method is a widely used and popular approach to analysing qualitative data in health research, however, there are some potential limitations that should be acknowledged. Whilst Framework Method offers a systematic approach to data analysis, the categorisation, organisation and interpretation of the data remains dependent on the knowledge and experience across the wider research team [58]. As such the researchers involved in all stages of analysis have an excellent track record of qualitative research and more specifically the application of Framework Method in applied health research [59–62]. It should finally be noted that no triangulation of data collection was performed.

## Conclusions

This research provides important findings regarding the mechanisms which influence and hinder breastfeeding practices across an ethnically diverse sample which can be used to help shape both existing and future breastfeeding services and interventions. Whilst all ethnic minority women held a strong intention and desire to breastfeed; there were clear barriers that influenced some mothers’ decision to introduce formula and/or stop breastfeeding earlier than planned. Whilst there were some commonalities across all mothers, the barriers and the strength of why they exist were often culturally located. For example, whilst lack of milk was disclosed to be a barrier for many mothers, this for South Asian mothers was mostly driven by weight concerns, particularly among those who had infants with a low birth weight, which was often met by pressure by their immediate family to introduce infant formula. This therefore highlights the need for culturally tailored interventions and services which not only acknowledge the traditional and familial practices of the mother and the important people that may influence her decision to breastfeed but to also understand the wider impact this may have on breast feeding practices.

In a bid to reduce health inequalities local health services must play an integral role in ensuring the needs of ethnically diverse mothers are being met and are culturally receptive to acknowledge the barriers as identified. This research highlights the importance of prenatal support, not only in advocating the benefits of breastfeeding but also to address the culturally specific misconceptions and barriers of breastfeeding. This study also revealed that whilst mothers valued the practical support offered from healthcare professionals, the access and engagement of these services appeared to vary across ethnic groups and locality. Knowledge and awareness alongside continuity of care, consistency of advice and developing a good relationship with the healthcare practitioner and were all viewed as important facilitators to with breastfeeding services and ultimately empowering them to succeed on their breastfeeding journey.

## Data Availability

The datasets generated and analysed during the current study are not publicly available due to confidentiality but are available from the corresponding author on reasonable request.

## Abbreviations

BAME: Black and Minority Ethnic
EU: European Union
FA: Framework Approach
